# Anti-Autophagy Mechanism of Zhi Gan Prescription Based on Network Pharmacology in Nonalcoholic Steatohepatitis Rats

**DOI:** 10.3389/fphar.2021.708479

**Published:** 2021-07-19

**Authors:** Chufeng Qin, Lichuan Luo, Yusheng Cui, Li Jiang, Beilei Li, Yijie Lou, Zhuofan Weng, Jingwen Lou, Chenxin Liu, Cuiting Weng, Zhaojun Wang, Yunxi Ji

**Affiliations:** ^1^ School of Basic Medical Sciences, Zhejiang Chinese Medical University, Hangzhou, China; ^2^ School of Humanities and Management, Zhejiang Chinese Medical University, Hangzhou, China; ^3^ The First School of Clinical Medicine, Zhejiang Chinese Medical University, Hangzhou, China; ^4^ Yuanben Health Management Co. LTD, Hangzhou, China; ^5^ Department of Traditional Chinese Medicine, Changan Hospital, Xian, China; ^6^ Department of General Practice, The First Affiliated Hospital of Zhejiang Chinese Medical University, Hangzhou, China

**Keywords:** network pharmacology, non-alcoholic steatohepatitis, zhi gan prescription, autophagy, molecular mechanisms

## Abstract

**Background and Aims:** Zhi Gan prescription (ZGP) has been clinically proven to exert a favorable therapeutic effect on nonalcoholic steatohepatitis (NASH). This study purpose to reveal the underlying molecular mechanisms of ZGP action in NASH.

**Methods:** Systematic network pharmacology was used to identify bioactive components, potential targets, and the underlying mechanism of ZGP action in NASH. High fat (HF)-induced NASH model rats were used to assess the effect of ZGP against NASH, and to verify the possible molecular mechanisms as predicted by network pharmacology.

**Results:** A total of 138 active components and 366 potential targets were acquired in ZGP. In addition, 823 targets of NASH were also screened. *In vivo* experiments showed that ZGP significantly improved the symptoms in HF-induced NASH rats. qRT-PCR and western blot analyses showed that ZGP could regulate the hub genes, PTEN, IL-6 and TNF in NASH model rats. In addition, ZGP suppressed mitochondrial autophagy through mitochondrial fusion and fission via the PINK/Parkin pathway.

**Conclusion:** ZGP exerts its effects on NASH through mitochondrial autophagy. These findings provide novel insights into the mechanisms of ZGP in NASH.

## Introduction

Non-alcoholic fatty liver disease (NAFLD) is a metabolic disorder caused by obesity, diabetes, and other factors that lead to fatty degeneration of the liver (Cheng et al., 2017). Based on the disease development, NAFLD can be defined as simple fatty liver (SFL), nonalcoholic steatohepatitis (NASH), hepatic fibrosis, liver cirrhosis, or hepatocellular carcinoma (HCC). NASH is a crucial step in NAFLD, and occurs between SFL and severe liver disease. The prevalence of NASH in the developed world is at least 2–3% (Satapathy and Sanyal, 2015). About 30% of patients with NAFLD are estimated to develop NASH (Younossi et al., 2016). However, NASH may progress to hepatic fibrosis, decompensated cirrhosis (DCC), and even HCC. Therefore, NASH is one of the most severe public health concerns worldwide. Early diagnosis and treatment can ameliorate disease progression in patients with NASH (Younossi et al., 2011; Dulai et al., 2017; Younossi et al., 2017).

The pathogenesis of NASH is complicated and has yet to be fully clarified. In recent years, the “multiple-hit” theory has highlighted the critical role of endoplasmic reticulum stress (ERS) in NAFLD, particularly in NASH. Hypoxia, oxidative stress, exogenous substances and the accumulation of unfolded proteins in the endoplasmic reticulum can disrupt endoplasmic reticulum homeostasis, leading to ERS, disturbances in hepatic lipid metabolism, inflammatory necrosis and apoptosis of hepatocytes, and ultimately the development of NASH (Ashraf and Sheikh, 2015; Buzzetti et al., 2016). It has been demonstrated that in NASH patients, accumulation of fatty acids elevates ERS (Lake et al., 2014; Zhang et al., 2014). ERS not only induces cytokine production, but also increases oxidative stress, mitochondrial dysfunction and inflammation, which is harmful to cell physiology and homeostasis (Victor et al., 2021). It has been demonstrated that both persistent ERS and mitochondrial dysfunction play a crucial role in the progression from NALFD to NASH (Malhi and Kaufman, 2011; Li et al., 2015). Therefore, inhibition of ERS may be the key to the treatment of NASH.

Zhi Gan prescription (ZGP) is composed of six herbal medicines (25 g of Codonopsis Radix, 20 g of Atractylodes Macrocephala Koidz., 20 g of Fructus Crataegi, 15 g of Polygoni Cuspidati Rhizoma Et Radix, 20 g of Cassiae Semen, and 10 g of Curcumaelongae Rhizoma, to a concentration of 1.1 g/ml). ZGP is an experiential prescription of Professor Fusheng Zhou for the treatment of NASH, which can tonify qi and strengthen the spleen, and dissipate blood stasis and phlegm (Ji et al., 2010). Clinical studies show that the curative effect of ZGP can efficaciously improve clinical symptoms and quality of life (Chen et al., 2007). However, the mechanisms of ZGP activity in NASH are not unclear.

Compared with western medicine, TCM (Traditional Chinese Medicine) is characterized by its multi-component and multi-target nature. In the therapy of disease, TCM exerts its effects on multiple biological processes *via* diverse bioactive components, which act on multiple targets (Liu et al., 2019). The emergence of network pharmacology has provided a new method to explore the complex network relationship between multi-components and multi-targets (Zhao et al., 2020). Network pharmacology is a branch of pharmacology that takes the theory of systems biology and multiple pharmacology as a foundation, and biomolecular network as the main means to identify the active compounds in herbal medicines and potential therapeutic targets (Hopkins, 2007).

In this study, we sought to identify the active ingredients, therapeutic targets, and the mechanism of action of ZGP. Potential therapeutic targets were identified by network pharmacology analysis, followed by the construction an herbal-chemical-protein-disease network and the identification of hub genes identification. *In vivo* experiments were performed to validate the potential molecular mechanisms. The result of this study provides novel insights into the mechanisms underlying the efficacy of herbal TCM in NASH and provide a reference for preparing ZGP for treating NASH.

## Materials and Methods

### Identification and Screening of the Chemical Ingredients in ZGP

ZGP contains six herbs; the compounds in these herbs are the ingredients of ZGP. These ingredients were collected from three databases: Traditional Chinese Medicine System Pharmacology (TCMSP, https://tcmspw.com/tcmsp.php) (Ru et al., 2014), Herbal Ingredients’ Targets Database (HIT, http://lifecenter.sgst.cn/hit) (Ye et al., 2011; Yang et al., 2013) and Traditional Chinese Medicines Integrated Database (TCMID, http://www.megabionet.org/tcmid) (Xue et al., 2013; Yang et al., 2013; Huang et al., 2018). In addition, the STITCH database (http://stitch.embl.de) was used to retrieve compound targets (Szklarczyk et al., 2016). After removing duplicates, 142 compounds and 4,131 targets were obtained.

TCM is generally provided as an oral preparation. It exerts biological effects upon reaching the organs and tissue after absorption, distribution, metabolism, and excretion (ADME) *in vivo* (Wang et al., 2012; Yang et al., 2015; Yang et al., 2018b). Poor ADME properties largely account for the failure of a drug to exert a pharmacodynamic act on the target site *in vivo* (Wang et al., 2012). Drug-like (DL) is an important parameter of ADME. The assessment of compound drug-likeness contributes to characterized the bioactive ingredients in TCM formulas (Li et al., 2012; Wang et al., 2012). In this research, the quantitative estimate of drug-likeness (QED) proposed by Bickerton was utilized to pre-selected compounds with pharmaceutical properties in ZGP (Bickerton et al., 2012).

### Screening for Compound-Related Targets

The core targets of ZGP can be verified by analyzing the target profile of ZGP ingredients. The target that interacts with the majority of compounds can be considered the core target for the pharmacological action of the formulation. In the meantime, the probability of a target was calculated by using a binomial statistical model (Liang et al., 2014; Yang et al., 2018a) as follows:

$$P\left(X\geq k\right)=\overset{n}{\underset{m=k}{\sum }}C_{n}^{m}{\left(p\right)}^{m}{\left(1-p\right)}^{n-m}$$

In this formula, *k* is the count of compounds interacting with the disquisitive target, *n* is the total number of compounds, and *p* denotes the ratio of the average number of compounds per target of the total number of compounds. *p* (*X* ≥ *k*), describes the probability of a target interacting with more than *k* compounds of *n* compounds in the profile by the random choice method and the value is adjusted by the false discovery rate method. These statistics contribute to evaluate the likelihood of the stochastic result. *p* ≤ 0.01 means that the actual quantity of compounds that the target interacts with is significantly greater than the expected, therefore, this target can be considered as a core target for the formula.

### Screening of NASH-Related Targets

NASH-related genes were screened and collected from Online Mendelian Inheritance in Man (OMIM, https://omim.org) (Amberger et al., 2019), DisGeNET (http://www.disgenet.org/) (Pinero et al., 2017), and GeneCards (https://www.genecards.org/) (Safran et al., 2010). DisGeNET is a comprehensive platform containing a large amount of genetic basis data associated to human disease. The keyword “non-alcoholic steatohepatitis” was searched in the platform and NASH-related genes were screened builded on a score ≥0.4. NASH-related genes with GeneCards Inferred Functionality Score (GIFtS) ≥52 were picked up from the GeneCards database. The target ID was matched with the gene symbol by searching in the UniProtKB (https://www.uniprot.org/) (Breuza et al., 2016). The network was constructed by Cytoscape (v3.8.0).

### Protein-Protein Interaction Network Constrution

NASH-related targets and ZGP corresponding targets were input into Venn 2.1 software to draw Venn diagrams and screen the common targets of both. Then, the resulting target information was input into STRING database to construct the PPI network. Next, we used CytoNCA (v2.1.6), a plug-in of Cytoscape, to evaluate the intersection.

### GO and KEGG Pathway Enrichment Analysis

In order to identify the functions of the formula targets and NASH-related genes, gene ontology (GO) enrichment analysis and Kyoto Encyclopedia of Genes and Genomes (KEGG) pathway analysis was performed. A hypergeometric test was preferable conducted to assess the relationship between annotation terms and the query genes, and the probability can be calculated as follows:

$$P=1-\sum_{i=0}^{k-1}\frac{\left(\begin{array}{c} M\\ i \end{array}\right)\left(\begin{array}{c} N-M\\ n-i \end{array}\right)}{\left(\begin{array}{c} N\\ n \end{array}\right)}$$

where, *N* is the total number of genes in the reference list, *M* denotes the number of genes annotated by GO or KEGG pathway, *n* represents the count of number of query genes, and *k* is the number of common genes between query genes and reference genes. According to the false discovery rate method, *p* < 0.01 demonstrates the significant association between annotated genes and query genes. In this study, GO enrichment analysis and KEGG pathway analysis was carried out for obtaining molecular function (MF), cellular component (CC), biological process (BP), and a functional pathway.

### Model

A total of 120 Sprague-Dawley rats (180–220 g) were provided by the laboratory animal center of Zhejiang Chinese Medical University. This animal research protocol was ratified by the Institutional Animal Care and Use Committee of the Zhejiang Chinese Medical University. All the Sprague-Dawley rats were bred in a lab room with a 12 h light:12 h dark cycle and controlled temperature at 24 ± 2°C and allowed ad libitum acquire to rodent chow and water.

The rats were randomly divided into six groups, including normal (*n* = 20), model (*n* = 20), positive medicine (*n* = 20), high-dose ZGP (*n* = 20), medium-dose ZGP (*n* = 20) and low-dose ZGP (*n* = 20) groups. Rats in the normal group were allowed free acquire to a standard diet. Animals in the model and treatment groups accepted a high-fat (HF) diet (2% cholesterol + 10% lard + 88% standard diet) for 8 weeks. After 8 weeks, rats in the high-dose ZGP, medium-dose ZGP, and low-dose ZGP groups were given a ZGP decoction at 22.88, 15.29, and 7.59 g/kg, respectively. Rats in the positive medicine control group received atorvastatin calcium tablets (1 mg/kg/day, Pfizer Inc., US) by gavage for 4 weeks. Rats in the normal and model groups were given distilled water equivalent to the ZGP.

### Histology

The right liver lobe was removed and cut a piece of liver tissue (no more than 2 mm thick) with a blade and plotting plane flat. The liver tissue was immersed in 10% neutral formalin solution for fixation (Jeon et al., 2014; Zhao et al., 2018), and then was implemented a series of steps of dehydrating, embedding in paraffin, cutting into 5 μm slices, and drying. Next, liver sections were subjected to hematoxylin-eosin staining, passed through a series of dehydrating with graded ethanol, clearing in xylene, and sealing in neutral gum. A low power microscope was used to observe liver sections and theoretically, the cytoplasm would be stained various shades of red, while the nucleus would be blue. The changes of fat, inflammatory cell infiltration, and balloon-like changes in lobules was observed under a 20-fold objective lens by selecting three visual regions randomly. Two inspectors assessed the degree of the lesion using a double-blind method, and then calculated the activity score of NAFLD (NAS) (Kleiner et al., 2005). The rules are shown in Table 1.

**TABLE 1:** The NAS of each group (x¯ ± *s*, n = 10).

Hepatic steatosis	Intralobular inflammation	Balloon degeneration
0: <5%	0: Nothing	0: Nothing
1: 5–33%	1: <2	1: Little
2: 34–66%	2: 2–4	2: Large
3: >66%	3: >4	

### Biochemical Measurements in Serum

Each blood specimen (3–5 ml) was centrifuged for 10 min (3,000 rpm, 4°C), the upper layer of which was then collected in a 1.5 ml EP tube. An automatic biochemical analyzer was utilized to determine changes in serum lipid levels and hepatic functions (TC, TG, LDL-C, HDL-C, ALT, and AST). MDA and 4-HNE contents were measured using an ELISA kit following manufacturer instructions (Cell Biolabs, San Diego, CA, United States).

### qRT-PCR

Experimental manipulations were performed as previously reported (Wang et al., 2015).

### Western Blot

For Western blot analysis, the experiments were proceed as previously described (Wang et al., 2020).

### Transmission Electron Microscopy

The removed cells and liver tissue were fixed by circulating with a fixation buffer on ice for 2 h, followed by six washing steps using 0.1 M sodium cacodylate and 3 mM calcium chloride on ice. The cells and liver slices approximately 80 μm were dissected using a Leica VT 1,000 s vibratome and then were postfixed with 1% osmium tetroxide, 0.8% potassium ferrocyanide, and 3 mM calcium chloride in 0.1 M sodium cacodylate for 1 h. Slices were washed three times with ice-cold distilled water and stained *en bloc* slices with 2% uranyl acetate at 4°C overnight. Stained slices were dehydrated with graded ethanol solutions, and then embedded in Durcupan ACM resin (Sigma). Ultrathin 80 nm liver sections were obtained using a Leica Ultracut UCT ultramicrotome, and poststained with uranyl acetate and lead salts. Subsequently, the digital images of slices were analyzed using a HITACHI H-7650 TEM under an 80 kV setting (Indicated magnification: ×25,000).

### Immunohistochemistry

Immunohistochemistry was performed according to the manufacturer’s instructions of the kits (Servicebio, China). Each slice was observed five visual regions randomly under high magnification (×200), and the Image-Pro Plus 6.0 was utilized to measure the integrated optical density (IOD) of each slice. The relative expressions of target proteins were represented by the average-optical density IOD/Area.

### Statistical Analysis

The data were presented as the means ± standard error. Statistical analysis was conducted by one-way ANOVA and followed by the Student’s t-test (for two groups) or Tukey’s test (for more than two groups). *p* < 0.05 were indicated statistically significant between groups.

## Results

### ZGP Compound-Target Network

A total of 142 components of ZGP were obtained from the TCMSP (v2.2, 2021.4), TCM-ID (2021.4) and HIT (2021.4) databases. These components were screened using the criteria of QED ≥0.2. In total, 138 bioactive components of ZGP were included; 46 of Codonopsis Radix, 24 of Atractylodes Macrocephala Koidz., seven of Crataegus pinnatifida Bge., 24 of Polygoni Cuspidati Rhizoma Et Radix, 19 of Cassiae Semen, and 22 of Curcumaelongae Rhizoma. Consequently, the compounds and their corresponding targets remained for subsequent analysis.

**FIGURE 1:**
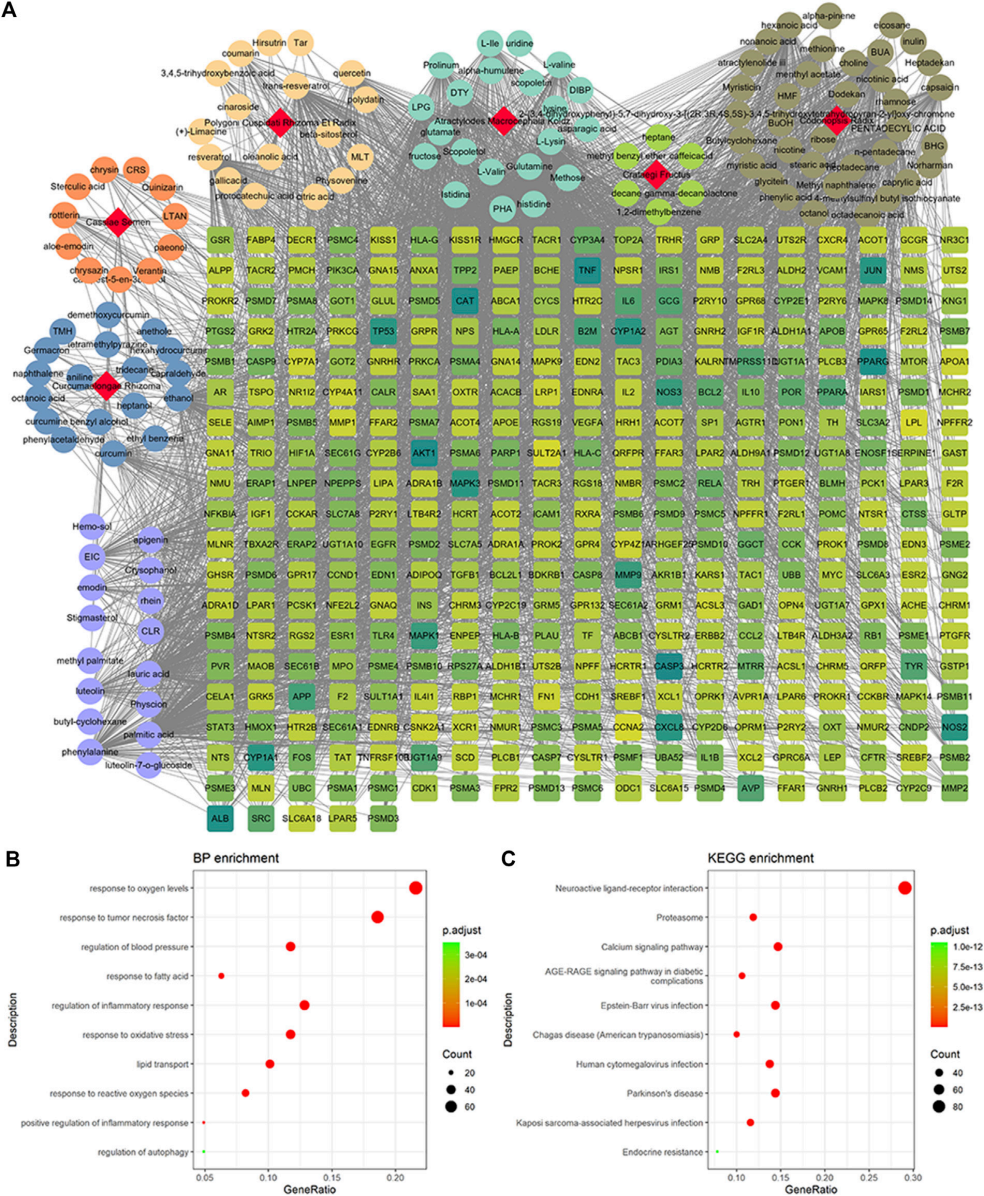
Potential targets of ZGP compounds. **(A)** ZGP compound-target network was generated by Cytoscape. **(B)** GO enrichment analysis for potential targets of ZGP. **(C)** KEGG pathway enrichment analysis for potential targets of ZGP.

A total of 4,131 targets for ZGP were obtained from the STITCH database (v5.0, 2021.4) with a DC ≥ 0.2. Then, each gene was scored by Materials and Methods 2.2, and 366 protein targets with a *p* ≤ 0.01 were selected as the formula targets. Information on the compounds with >10 targets, namely the main active components of ZGP, are listed in Supplementary Table S1. Among the major active compounds, palmitic acid exhibited a total of 601 targets, whereas resveratrol exhibited 332 targets, lauric acid 305 targets, hexanoic acid 274 targets, quercetin 133 targets, apigenin 85 targets, luteolin 77 targets. Citric acid, coumarin, curcumin, chrysin, phenylic acid, myristicin, stigmasterol demonstrated 70, 59, 58, 38, 19, 12, and 10 targets, severally. The corresponding association between compounds and targets is shown in Figure 1A. In addition, of the 138 bioactive components, the top 12 core components considered to be the most effective for NAFLD were luteolin, apigenin, myricetin, capsaicin, quercetin, oleanolic acid, beta-sitosterol, resveratrol, polydatin, curcumin, emodin, and scopoletin. These 12 core compounds are detailed in Table 2.

**TABLE 2:** The top 12 core compounds of ZGP.

PubChem cid	Molecule name	Formula	CAS number	Structure
5280445	Luteolin	C_15_H_10_O_6_	491-70-3	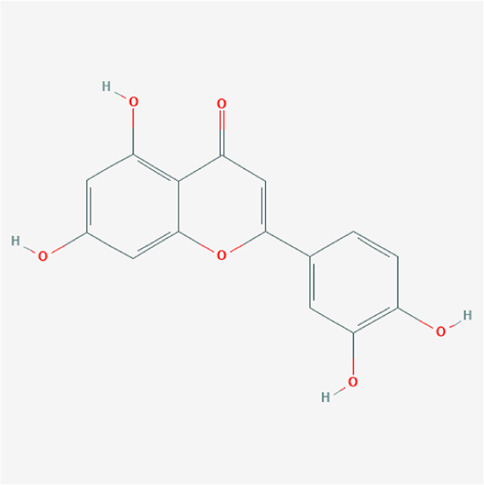
5280443	Apigenin	C_15_H_10_O_5_	520-36-5	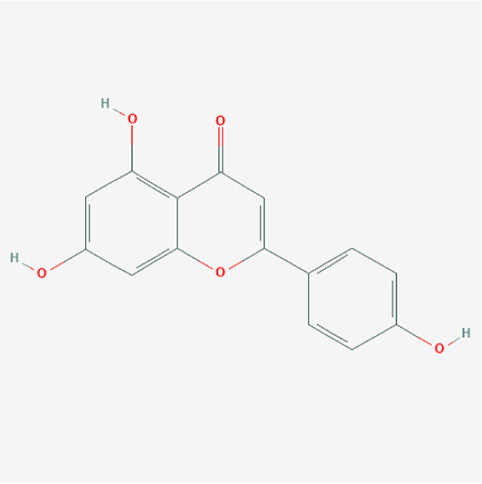
4276	Myristicin	C_11_H_12_O_3_	607-91-0	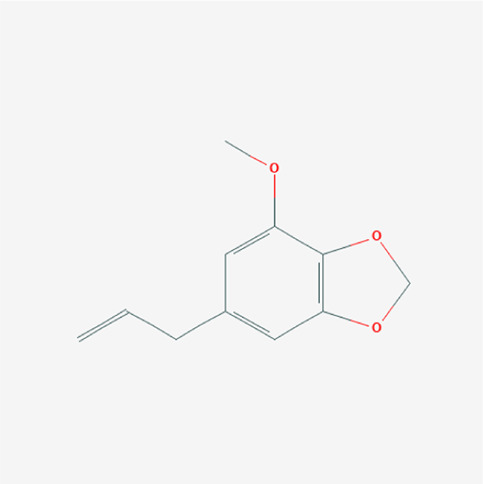
1548943	Capsaicin	C_18_H_27_NO_3_	404-86-4	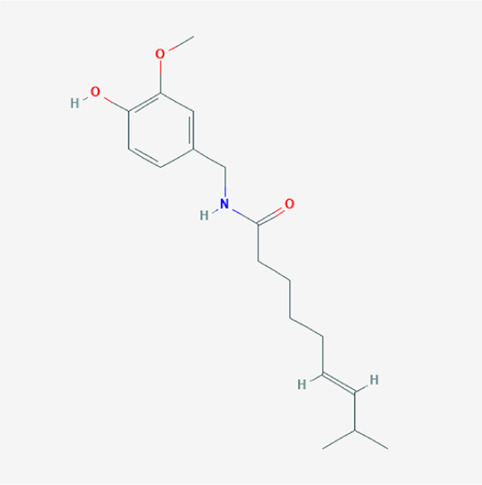
5280343	Quercetin	C_15_H_10_O_7_	117-39-5	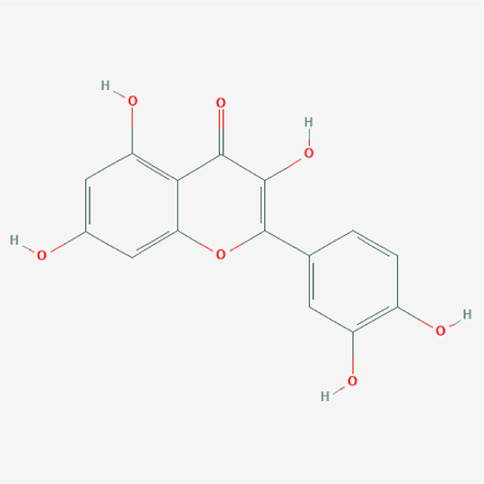
10494	Oleanolic acid	C_30_H_48_O_3_	508-02-1	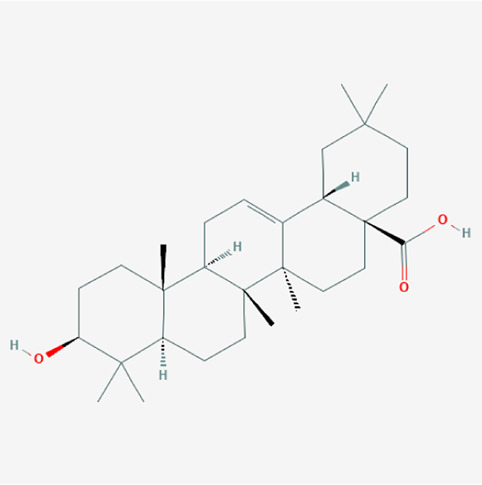
222284	Beta-sitosterol	C_29_H_50_O	83-46-5	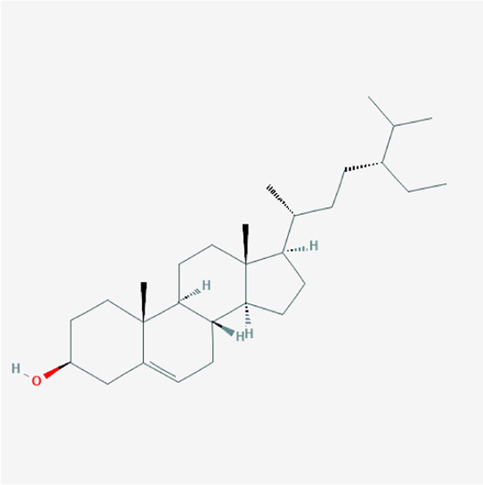
445154	Resveratrol	C_14_H_12_O_3_	501-36-0	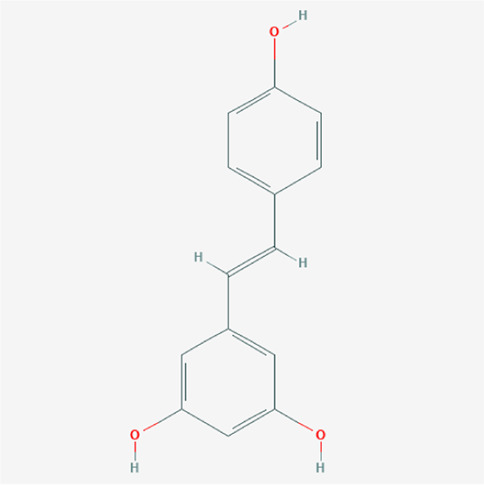
5281718	Polydatin	C_20_H_22_O_8_	65914-17-2	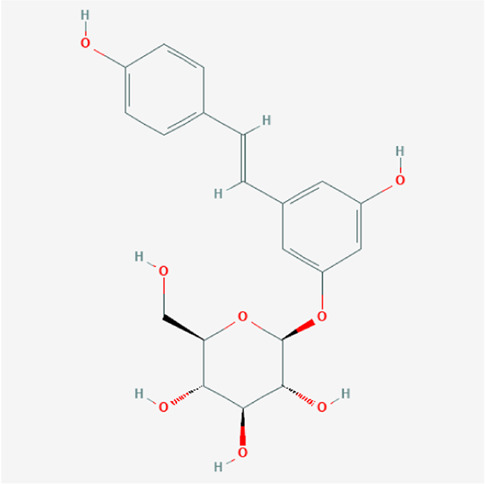
969516	Curcumin	C_21_H_20_O_6_	458-37-7	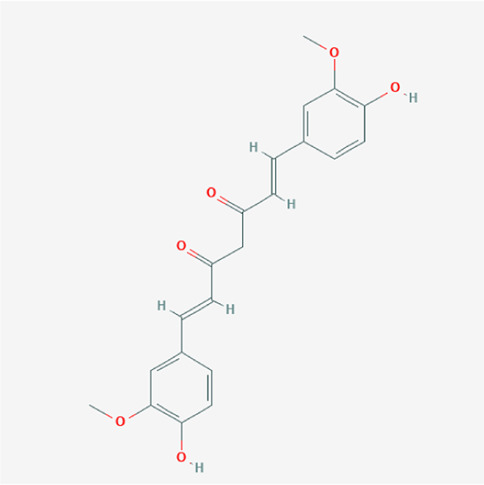
3220	Emodin	C_15_H_10_O_5_	518-82-1	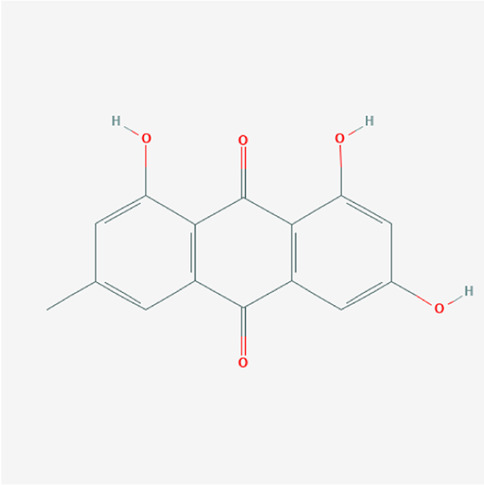
5280460	Scopoletin	C_10_H_8_O_4_	92-61-5	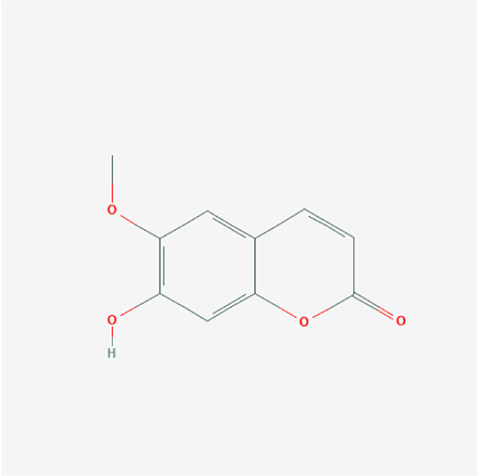

GO and KEGG pathway enrichment analyses were executed clarify the characteristics of targets for the key active components of ZGP (Figures 1B,C). These targets were discovered to be involved in biological processes, such as response to oxygen levels, response to tumor necrosis factor, regulation of blood pressure, response to fatty acids, regulation of the inflammatory response, response to oxidative stress, lipid transport, response to reactive oxygen species, positive regulation of inflammatory response, and regulation of autophagy. The top 10 pathways included neuroactive ligand-receptor interaction, proteasome, calcium signaling pathway, AGE-RAGE signaling pathway in diabetic complications, Epstein-Barr virus infection, Changas disease, human cytomegalovirus infection, Parkinson’s disease, Kaposi sarcoma-associated herpesvirus infection and endocrine resistance.

### Network of NASH-Related Targets

**FIGURE 2:**
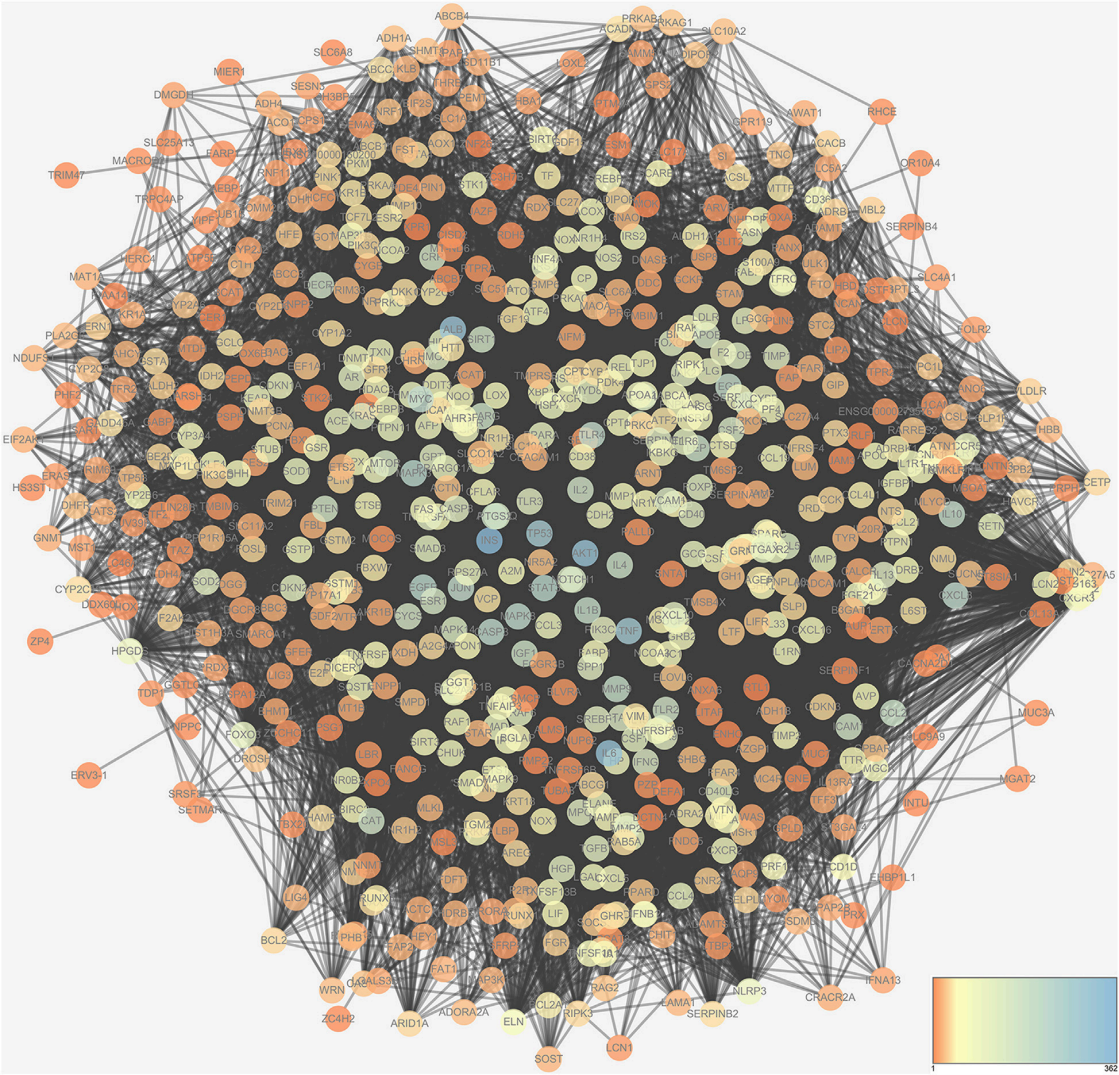
NASH-target network. The NASH-target network was generated by Cytoscape 3.6.0 software.

NASH occurrence and development are correlated to the regulation of multiple genes. Research on the relationship between the gene and gene-environment interactions can help to elucidate the pathogenesis of NASH. In this study, 1,551 NASH-related targets were collected from the OMIM (2021.4), DisGeNET (v7.0, 2021.4), and GeneCards (v5.2, 2021.4) database. The network of NASH-related genes containing 711 nodes and 17,280 edges is shown in Figure 2.

### ZGP-NASH PPI Network

**FIGURE 3:**
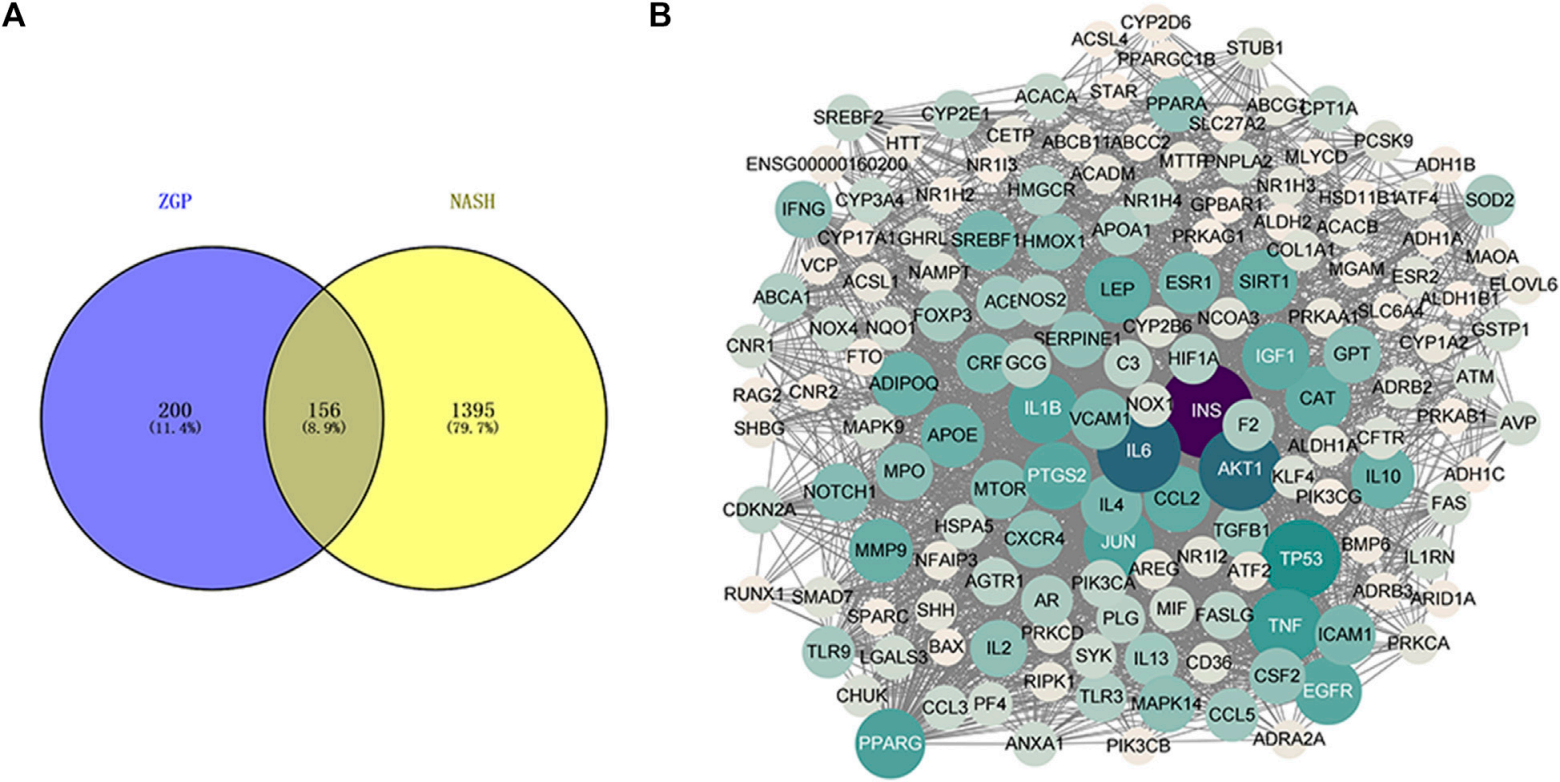
**(A)** Venn diagram of targets for ZGP treating NASH. **(B)** ZGP-NASH PPI network.

A Venn diagram was constructed to acquire the common targets of the ZGP bioactive component targets and the NASH-related targets (Figure 3A). The acquired genes were the ZGP targets in the treatment of NASH. These targets were introduced into Cytoscape to construct PPI network (Figure 3B).

### GO and Pathway Enrichment Analysis

**FIGURE 4:**
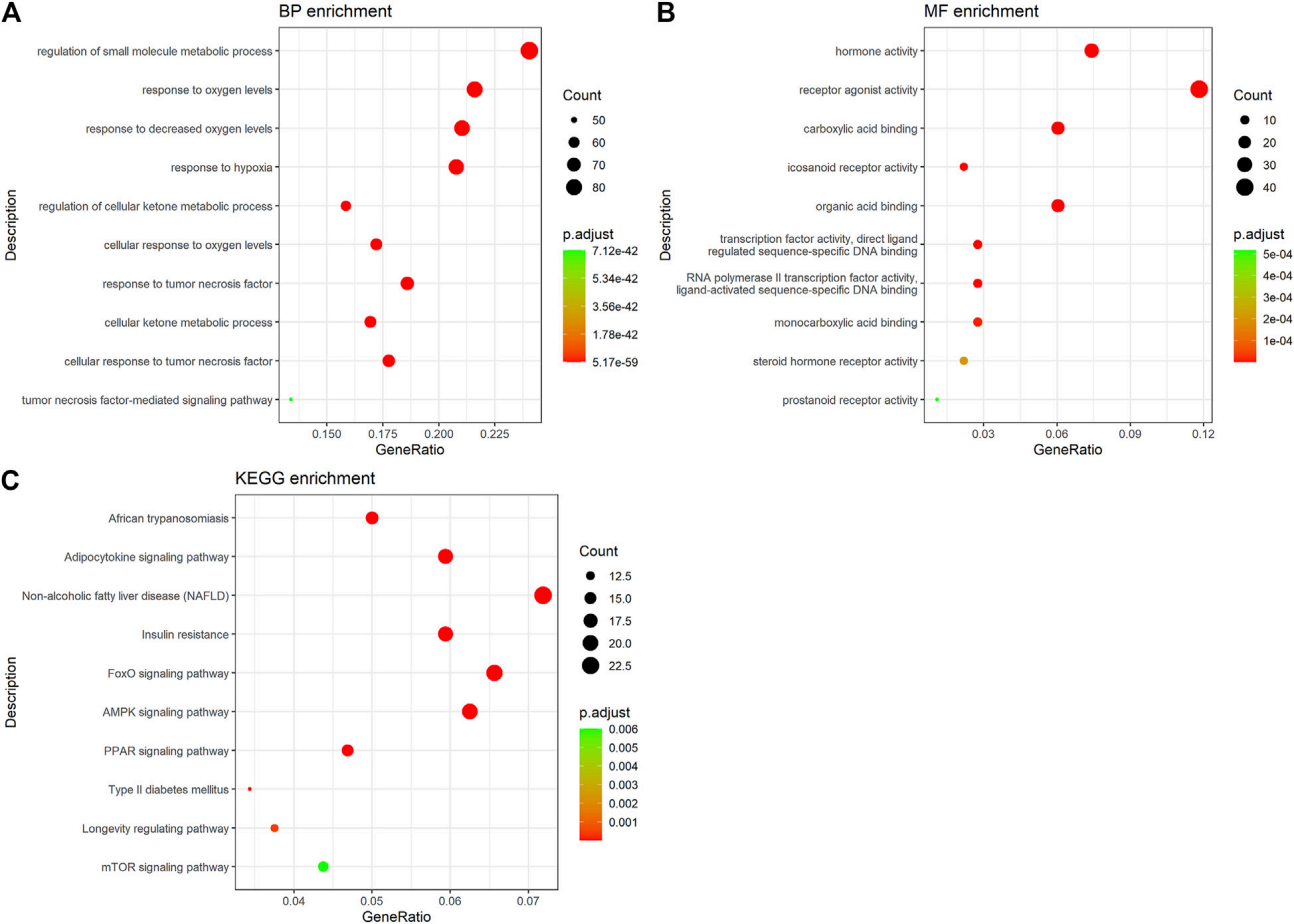
GO and KEGG enrichment analysis of hub targets. **(A)** GO-BP enrichment analysis of hub targets. **(B)** GO-MF enrichment analysis of hub targets. **(C)** KEGG pathway enrichment analysis of hub targets.

Go enrichment analysis was performed to identify common features of the formula targets and disease genes in terms of GO annotation, respectively. The number of GO terms that are significantly associated with the formula targets for MF, BP, and CC was 2,424, 178, and 119 (*p* < 0.01), respectively. By comparing with the results of disease genes, we found that 1,158 and 61 GO were terms significantly associated with the disease targets for MF and BP, respectively. We selected the common GO terms shown in Figures 4A,B. The results for BP showed that these targets are involved in regulating the metabolism of small molecules, response to oxygen levels, response to lessened oxygen levels, response to hypoxia, regulation of cellular ketone metabolic process, cellular response to oxygen levels, response to tumor necrosis factor, cellular ketone metabolic process, cellular response to tumor necrosis factor, and the tumor necrosis factor-mediated signaling pathway (Figure 4A). They exhibited various molecular functions, including hormone activity, receptor agonist activity, carboxylic acid binding, icosanoid receptor activity, organic acid binding, transcription factor activity direct ligand regulated sequence-specific DNA binding, RNA polymerase II transcription factor activity ligand-activated sequence-specific DNA binding, monocarboxylic acid binding, steroid hormone receptor activity, and prostanoid receptor activity (Figure 4B).

Enrichment analysis of the KEGG pathway showed that the active ingredients of ZGP had a significant effect on the pathway (*p* < 0.05). The top pathways (count number ≥10) included African trypanosomiasis, the adipocytokine signaling pathway, NAFLD, insulin resistance, the FoxO signaling pathway, the AMPK signaling pathway, the PPAR signaling pathway, type II diabetes mellitus, the longevity regulating pathway, and the mTOR signaling pathway (Figure 4C and Supplementary Figure S1). The targets of the main active components of ZGP were distributed in different metabolic pathways. The mutual regulation of “multi-components, multi-targets, and multi-pathways” is a possible mechanism of NASH treatment.

### Alleviation of Pathological Changes in the Liver Following ZGP Treatment

**FIGURE 5:**
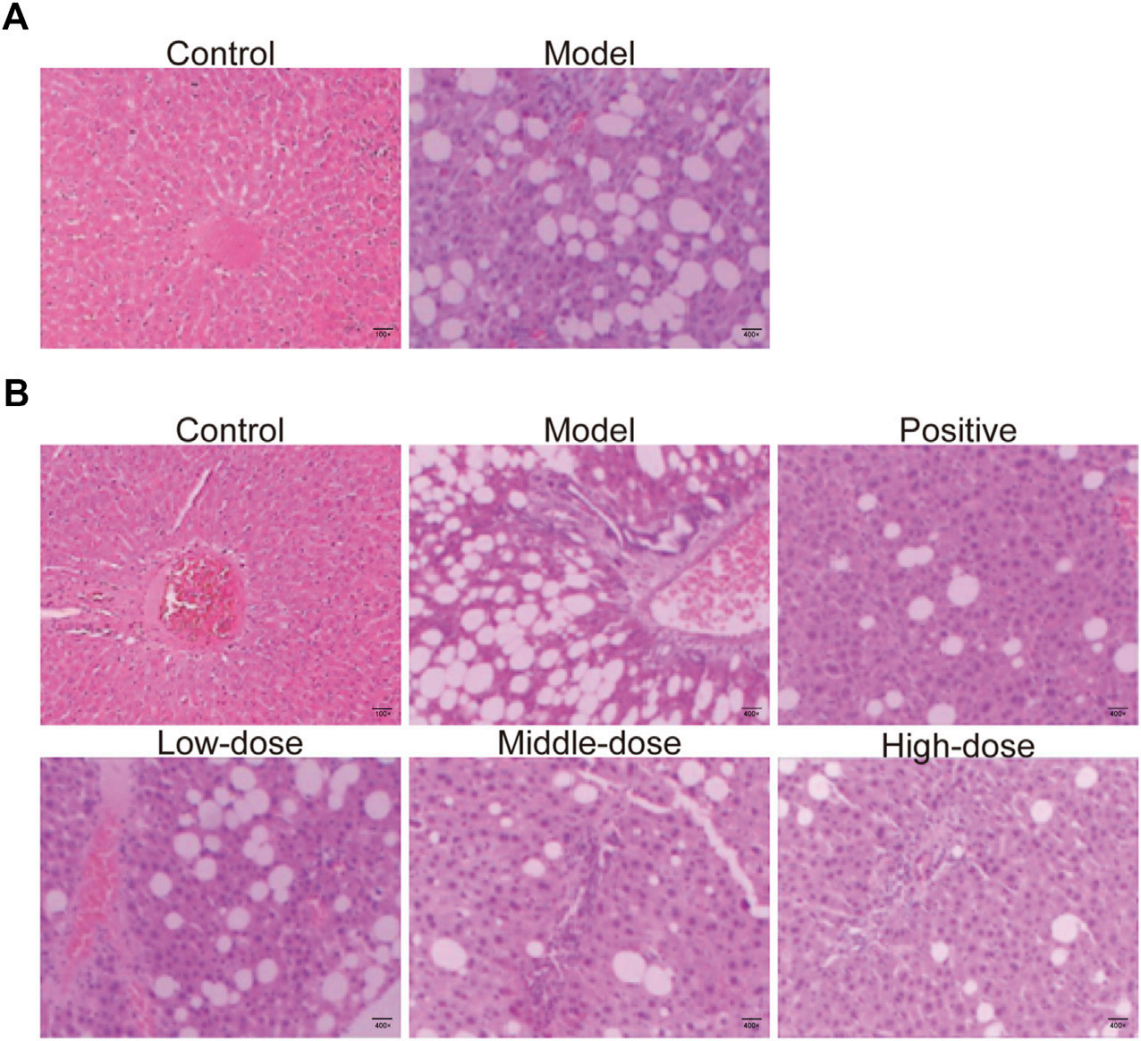
HE-staining of liver tissue. **(A)** HE-staining of liver tissue from rats fed with 8 weeks in control and model groups. **(B)** HE-staining of liver tissue of rats fed with 12 weeks in control, model, positive medicine, and ZGP-treated groups.

Liver tissues obtained from animals in all groups were stained with HE, as shown in Figure 5A. Liver sections from animals in control group presented normal lobular architecture, and liver cells with well-preserved cytoplasm and well-defined nucleus. However, typical vesicular steatosis, hepatocellular ballooning, and lipid droplets were observed in tissue from the NAFLD model group after 8 weeks of feeding with a HF diet. This suggests that we successfully established an animal model of NAFLD.

After 4 weeks of treatment with normal saline, ZGP or atorvastatin, the livers were collected from rats in all groups and analyzed using HE stain. The structure of the liver lobules was unclear in animals from the model group. The radially disposed hepatic cord was not obvious. Most cells were swollen, varied in size, and the edge was fuzzy. The cytoplasm was filled with different-sized fat vacuoles that pushed the nucleus to the periphery, and there were significant changes in fat, particularly in the hepatic lobule exchange, as well as different levels of inflammatory infiltration and liver cell debris-like necrosis (Figure 5B). Different doses of ZGP reduced the liver lesions by varying degrees, and this was most significant for animals in the high-dose ZGP group (Figure 5B). High-dose ZGP significantly decreased steatosis, hepatocyte ballooning, and lobular inflammation compared to the model group.

### Effect of ZGP on Liver Inflammation Score

The NAS of the model group was 7.98, which was higher than that of the control group; therefore, NASH could be diagnosed according to the scoring criteria. In the high-dose ZGP group, NAS decreased significantly by more than 50% compared with that of the model group (Table 3).

**TABLE 3:** The NAS of each group (x¯ ± *s*, n = 10).

Group	NAS
Control	0.00 ± 0.00
Model	7.98 ± 0.02
Positive	3.70 ± 0.46
Low-dose	7.99 ± 0.01
Middle-dose	3.68 ± 0.30
High-dose	1.90 ± 0.01

### Effect of ZGP on Biochemical Parameters

**FIGURE 6:**
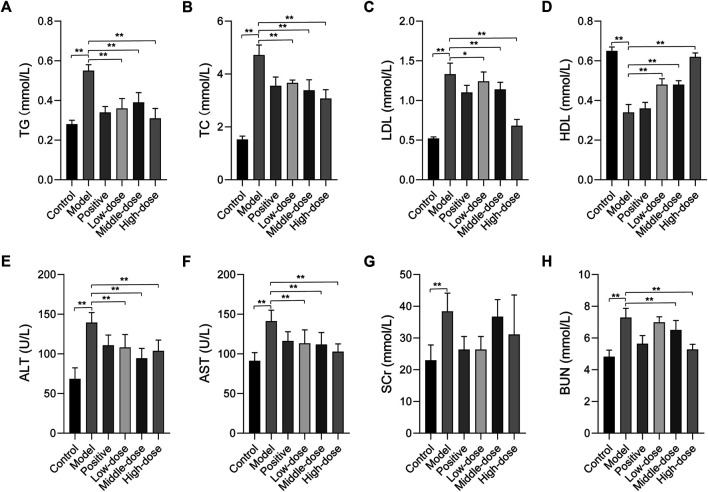
The effect of ZGP on biochemical parameters. **(A)** TG. **(B)** TC. **(C)** LDL. **(D)** HDL. **(E)** ALT. **(F)** AST. **(G)** SCr. **(H)** BUN. At least three repeats were performed, and the mean ± SD is presented, **, *p* < 0.01.

To determine the effect of ZGP on lipid metabolism, serum levels of TG, TC, LDL, and HDL were measured. In contrast to the normal control group, the levels of TG, TC, and LDL were significantly increased, while those of HDL were markedly decreased in the model group (Figures 6A–D). However, 4 weeks of treatment with ZGP and positive medicine significantly decreased the levels of TG, TC, and LDL, and increased the levels of HDL compared to the model group. The results were consistent between animals who received the middle-dose of ZGP and those who received the positive control. High-dose ZGP almost restored the levels of TG, TC, LDL, and HDL to those observed in the normal group. This suggested that ZGP can regulate lipid metabolism.

The serum levels of ALT and AST were determined to evaluate liver function. As shown in Figures 6E,F, the levels of ALT and AST were significantly elevated in animals in the model group compared to those in the normal control group; these levels were all markedly reduced after 4 weeks of treatment with ZGP and positive medicine compared to the model group. These data manifest that ZGP can effectively improve liver function.

We also evaluated the serum levels of SCr and BUN. The results suggested that SCr and BUN levels were significantly elevated in animals in the model group compared to those in the normal control group (Figures 6G,H). However, these were no significant changes in SCr levels. Furthermore, after 4 weeks of treatment with ZGP and positive medicine, the levels of BUN decreased compared to those in the model group. These data indicate that ZGP improves kidney function.

### Effect of ZGP on the Expression of Hub Targets

**FIGURE 7:**
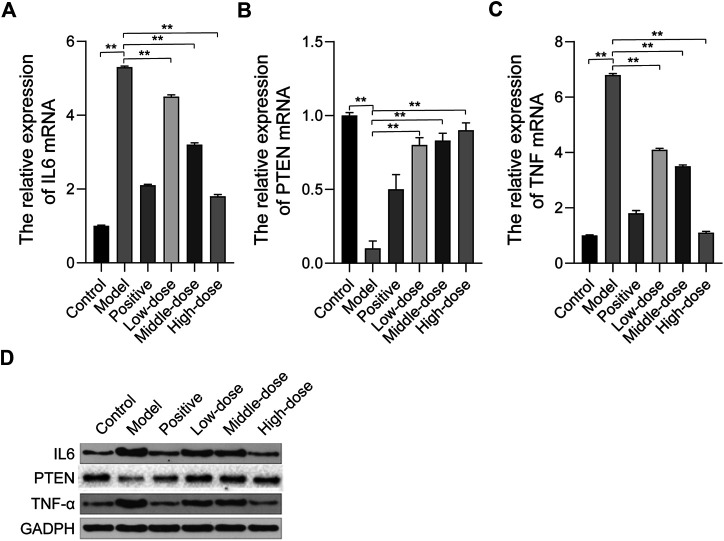
The effect of ZGP on the expression of hub targets. **(A-C)** The relative mRNA expression of IL6, PTEN, TNF were detected by qRT-PCR. **(D)** The protein expression of IL6, PTEN, TNF-α were detected by western blot. At least three repeats were performed, and the mean ± SD is presented, **, *p* < 0.01.

Based on the network pharmacology analysis, we examined the expression of hub targets by qRT-PCR and western blot, including phosphatase and tensin homolog (PTEN), interleukin 6 (IL6), and tumor necrosis factor-α (TNF-α). The qRT-PCR results showed that PTEN mRNA expression was down-regulated while IL6 and TNF-α mRNA expression was up-regulated in model rats. After 4 weeks of treatment with ZGP, PTEN mRNA expression increased, while IL6 and TNF-α mRNA expression decreased (Figures 7A–C). Western blot was used to detected the protein expression levels of PTEN, IL6 and TNF-α, the results are consistent with qRT-PCR results (Figure 7D). These results suggested that ZGP can improve NASH by regulating these hub genes, supporting the network pharmacology results.

### Effect of ZGP on Hepatic Oxidative Stress

**FIGURE 8:**
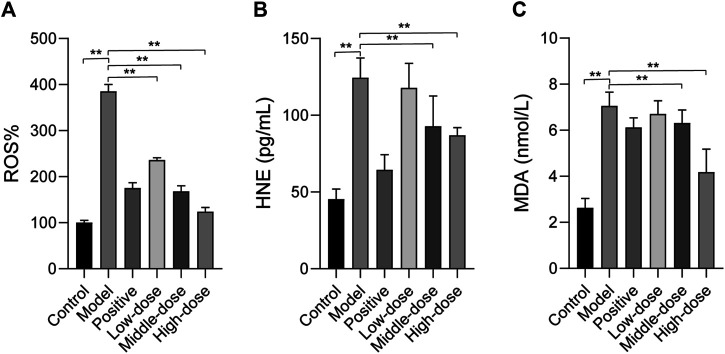
The effect of ZGP on hepatic oxidative stress. ZGP could regulate the level of ROS. **(A)**, and the contents of HNE. **(B)** and MDA. **(C)**. At least three repeats were performed, and the mean ± SD is presented, **, *p* < 0.01.

We observed GO terms for ZGP targets related to oxidative stress and reactive oxygen species. Hepatic mitochondrial ROS has a pivotal role in the development of NASH [18]. Oxidative stress can cause hepatocellular damage and death. The occurrence and development of liver injury can be induced by ROS, which promotes lipid peroxidation (Solmaz et al., 2020). Hence, we examined ROS levels to determine whether ZGP could reduce the level of oxidative stress in the liver. As shown in Figure 8A, ROS levels in model rats were obviously higher than those in the normal control group. Importantly, ROS level were reduced following treatment with ZGP, particularly at a high dose.

The levels of HNE and MDA, products of lipid peroxidation, were quantified in the liver. We also evaluated the expression of HNE and MDA. The results showed that HNE and MDA expression were significantly increased compared to the control (Figures 8B,C). HNE and MDA expression was down-regulated in the treatment groups, particularly with high-dose ZGP.

### ZGP Suppresses Liver Autophagy in Rats

**FIGURE 9:**
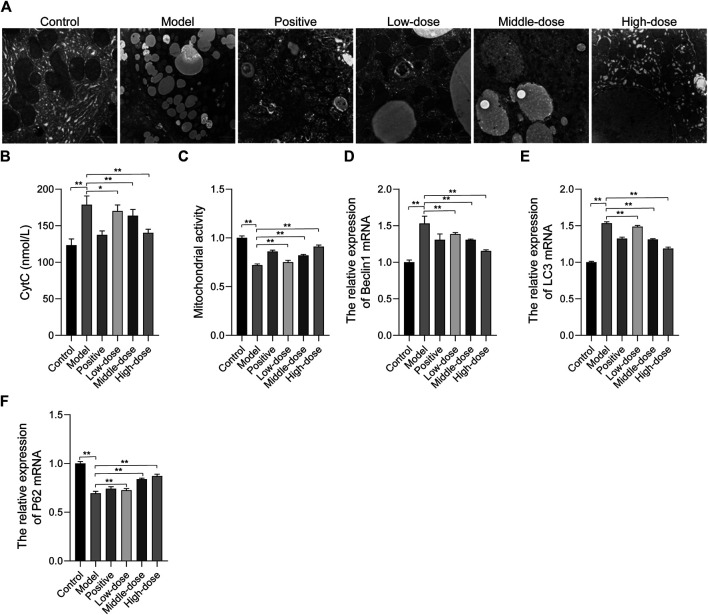
The effect of ZGP on mitochondrial autophagy. **(A)** Electron micrograph of ultra-thin sections of liver tissue. **(B)** CytC contents. **(C)** Mitochondrial activity. **(D)** Relative expression of Beclin1 mRNA. **(E)** Relative expression of LC3 mRNA. **(F)** Relative expression of P62 mRNA. At least three repeats were performed, and the mean ± SD is presented, **, *p* < 0.01.

Transmission electron microscopy was used to analyze the morphology of liver cells and lipid accumulation. In the control group, there were fewer lipid vacuoles with normal mitochondria and endoplasmic reticulum morphology compared with the other group. Abundant lipid droplets can be seen in the model tissues, the ultrastructure of liver cells has changed significantly, chromatin condenses on the edges, the endoplasmic reticulum expands, and mitochondria contract. Additionally, we found a significantly high level of autophagic vacuoles in tissue from the model group. Cellular integrity was maintained and lipid vacuoles were decreased in the treatment groups. ZGP significantly inhibited lipid accumulation and the structure in the liver appeared normal, especially in animals from the high-dose group (Figure 9A). We also found that the CytC content increased and the mitochondrial activity decreased in tissue from the model group, compared with that from the control group. Furthermore, in the treatment groups, there was a significant down-regulation of CytC and up-regulation of mitochondrial activity (Figures 9B,C).

We determined the level of autophagy in mice by assessing the conversion of LC3 from cytosolic LC3-I to LC3-II, as well as the levels of Beclin1 and p62. The results of qRT-PCR revealed that Beclin1 and LC3 expressions was increased, while the expression of p62 was reduced in the model group, compared with the control (Figures 9D–F), indicating that autophagy was increased in animals in the model group. In the treatment groups, Beclin1 and LC3 expressions was decreased, while p62 expression was increased.

### ZGP Regulates Mitochondrial Fusion and Fission

**FIGURE 10:**
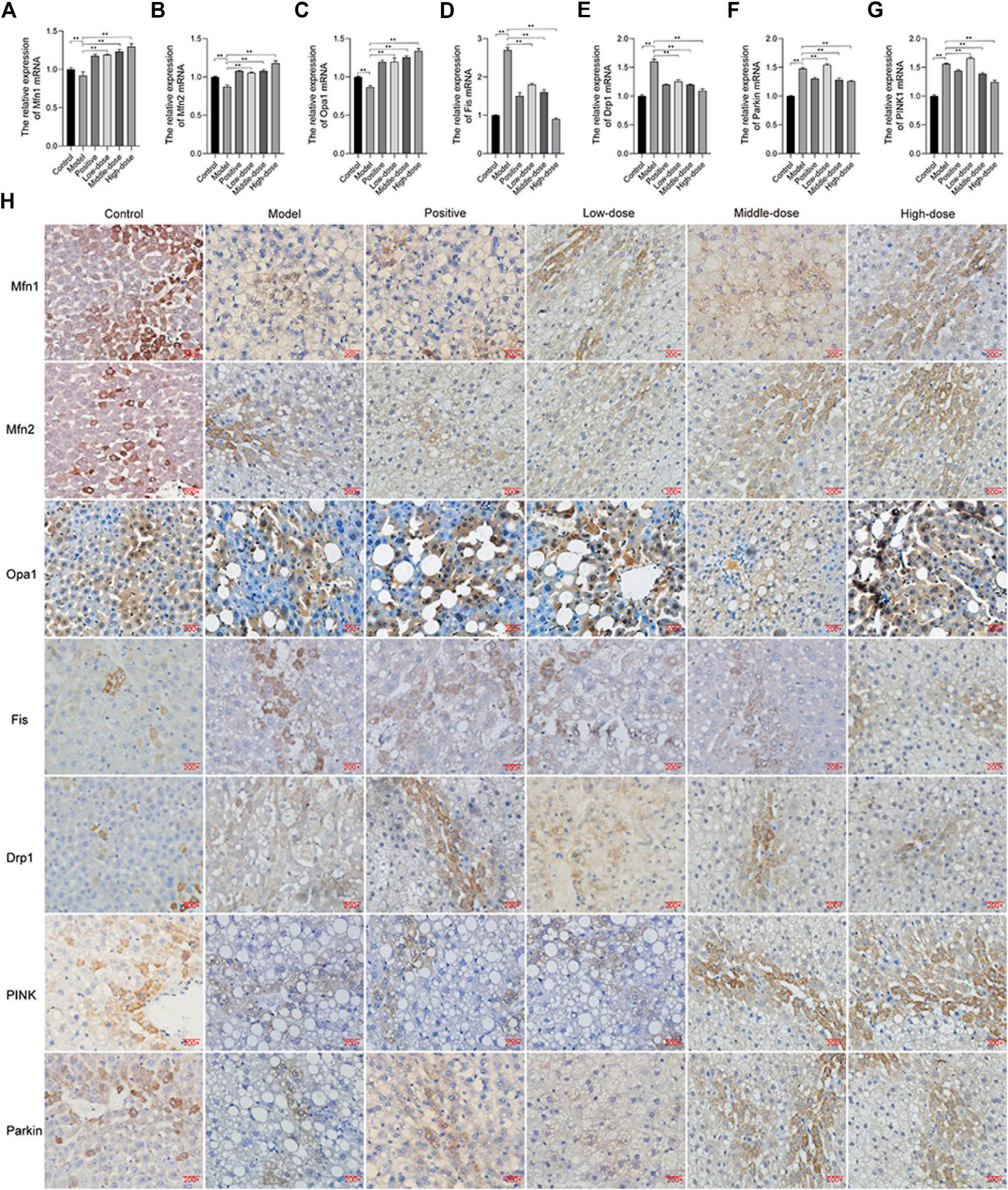
ZGP regulates mitochondrial fusion and fission. Relative expression of Mfn1. **(A)**, Mfn2 **(B)**, Opa1 **(C**), Fis **(D)**, Drp1 **(E)**, PINK1 **(F)** and Parkin **(G)** mRNA. **(H)** Immunohistochemistry of Mfn1, Mfn2, Opa1, Fis, Drp1, PINK1, and Parkin. At least three repeats were performed, and the mean ± SD is presented, **, *p* < 0.01.

We examined the expression of Mfn1, Mfn2, Opa1, Fis, and Drp1 by immunohistochemistry and qRT-PCR. The results showed that the expression of Mfn1, Mfn2, and Opa1 was down-regulated, while that of Fis and Drp1 was up-regulated in the model group, compared with the control (Figure 10). Treatment, particularly with a high-dose of ZGP, was able to induce the expression of Mfn1, Mfn2, and Opa1, and suppress the expression of Fis and Drp1 (Figure 10).

We also found that PINK1 and Parkin expression was increased in animal in the model group, compared with those in the control (Figure 10). Thus, ZGP could decrease the expression of PINK1 and Parkin.

## Discussion

Chinese medicine is currently the most widely used form of complementary and alternative medicine. It is holistic in its effects and works on multiple targets, through multiple channels. ZGP is a Chinese herbal medicine that has been proven to improve NASH liver damage. In recent years, network pharmacology research on disease treatment has attracted widespread attention. Based on modern computer simulation technologies, this method is an effective method for screening and predicting drug targets. In the present study, network pharmacology was used to explore the material basis and mechanism of ZGP in the treatment of NASH. TCM monomers were selected based on the common signal pathway, and the PPI network and transcription factor prediction networks were constructed. In addition, we performed animal experiments to confirm that ZGP could improve NASH.

*In vivo* studies were performed using a rat model of HF-induced NASH, which has been widely used in researched related to NASH (Chen et al., 2018; Mohamed et al., 2019). Pathological analysis revealed that ZGP significantly improved the physiological state of liver tissue. Moreover, ZGP can significantly reduce the levels of TG, TC, LDL, ALT, and AST in the serum of NASH rats. Thus, the results of the *in vivo* studies described herein confirmed that ZGP was able to improve lipid metabolism and reduce liver inflammation.

In this study, we identified three hub genes: PTEN, IL-6, and TNF-α. The tumor suppressor gene PTEN plays a key role in the development of steatosis, steatohepatitis, and fibrosis. Recent studies have shown that dysregulated expression of PTEN in hepatocytes, rather than PTEN mutations/deletions, is essential for the occurrence of NAFLD (Han et al., 2019). Pro-inflammatory cytokines/chemokines, such as TNF-a and IL-6, play a critical role in the progression of NAFLD to more advanced stages of liver damage (Cai et al., 2016). The results of qRT-PCR and western blot analyses showed that ZGP could restore the expression of PTEN, IL-6, and TNF-α, supporting the results of network pharmacology. Thus, ZGP was able to improve liver injury by regulating the expression of inflammatory factors.

ERS and mitochondrial dysfunction can lead to intracellular production of reactive oxygen species (ROS), which in turn aggravates mitochondrial damage and apoptosis (Haynes et al., 2004). We retrieved many items relating to oxidative stress and autophagy in the GO analysis of ZGP. Oxidative stress may be triggered by excessive levels of ROS. Inflammation is important for the progression of NAFLD to NASH, where ROS may promote hepatic insulin resistance and necrotic inflammation (Farzanegi et al., 2019). In addition, oxidative stress causes hepatic inflammation and fibrosis (Hu et al., 2018). In liver steatosis, mitochondrial dysfunction and oxidative stress promote the development of NASH. Thus, we examined the levels of ROS, and the contents of HNE and MDA; these were all increased in model rats. We found that ZGP could down-regulate the levels ROS and the contents of HNE and MDA, suggesting that ZGP is able to alleviate oxidative stress and reduce lipid peroxidation.

Autophagy is a basic catabolic process that degrades abnormal or unnecessary cell components through lysosomal activity, thereby improving cell survival (Lin et al., 2013). During autophagy, the targeted cytoplasmic component is located in the autophagosome, which is a double-membrane vesicle. Under disease conditions, autophagy acts as an adaptive response to stress, promoting survival in some cases, and cell death and morbidity in other cases (Kim et al., 2016). In the process of autophagy, LC3-I is coupled to phosphatidylethanolamine and recruits LC3-II to autophagosomal membranes, while p62 is a ubiquitin that interacts with LC3 and is degraded (Wu et al., 2018). Beclin1 plays an important role in mediating the localization of other autophagy proteins to the pre-autophagosomal structure (Leonard et al., 2019). Mitochondrial fusion is mainly controlled by three GTPase proteins: Mfn1 and Mfn2 in the outer membrane of mitochondria and Opa1 in the inner membrane of mitochondria. Mitochondrial division is mediated by dynamic like protein (Drp1), which is mainly located in the cytoplasm and is recruited into mitochondria upon division. This recruitment also depends on mitochondrial fision1 (Fis1). Our results suggested that ZGP could regulate the mitochondria *via* the expression of related proteins.

In conclusion, using network pharmacology, we show that ZGP acts on NASH through multi-compounds, multi-targets and multi-pathways. Furthermore, ZGP decreased the serum levels of TG, TC, LDL, ALT, and AST in NASH rats, and suppressed the levels oxidative stress. ZGP inhibited autophagy by mitochondrial fusion and fission. These findings provide novel insights into the mechanisms underlying the effects of ZGP in NASH.

## Data Availability

The original contributions presented in the study are included in the article/Supplementary Material, further inquiries can be directed to the corresponding authors.

## Glossary

ADME: absorption, distribution, metabolism, and excretion
ALOGP: octanol-water partition coefficient
ALERTS: structural alerts
AROMs: aromatic rings
BP: biological process
BSA: bovine serum albumin
CC: cellular component
DCC: decompensated cirrhosis
DL: drug-like
DAB: diaminobenzidine
ERS: endoplasmic reticulum stress
GIFtS: genecards inferred functionality score
GO: gene ontology
GAPDH: glyceraldehyde 3-phosphate dehydrogenase
HCC: hepatocellular carcinoma
HF: high fat
HIT: herbal ingredients’ targets database
HBDs: hydrogen bond donors
HBAs: hydrogen bond acceptors
IOD: integrated optical density
KEGG: kyoto encyclopedia of genes and genomes
MF: molecular function
NASH: nonalcoholic steatohepatitis
NAFLD: non-alcoholic fatty liver disease
NAS: activity score of NAFLD
OMIM: online mendelian inheritance in man
PVDF: polyvinylidene fluoride
PSA: molecular polar surface area
PBS: phosphate buffer saline
QED: quantitative estimate of drug-likeness
ROTBs: rotatable bonds
ROS: reactive oxygen species
SFL: simple fatty liver
TCM: traditional chinese medicine
TCMSP: traditional chinese medicine system pharmacology
TCMID: traditional chinese medicines integrated database
ZGP: zhi gan prescription.
