# Supplementary figures and images for: Anti-Autophagy Mechanism of Zhi Gan Prescription Based on Network Pharmacology in Nonalcoholic Steatohepatitis Rats
Source: Front Pharmacol. 2021 Jul 19;12:708479. doi: 10.3389/fphar.2021.708479 (PMC8326404; doi:10.3389/fphar.2021.708479)

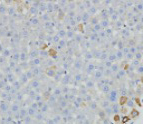

Supplement: Supplementary file 1 [file DataSheet1.ZIP › FigShares/FigShares/Figure 10/Fig 9H. Drp1/Control.jpg]

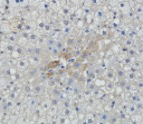

Supplement: Supplementary file 1 [file DataSheet1.ZIP › FigShares/FigShares/Figure 10/Fig 9H. Drp1/High.jpg]

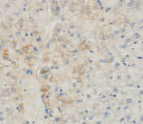

Supplement: Supplementary file 1 [file DataSheet1.ZIP › FigShares/FigShares/Figure 10/Fig 9H. Drp1/Low.jpg]

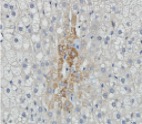

Supplement: Supplementary file 1 [file DataSheet1.ZIP › FigShares/FigShares/Figure 10/Fig 9H. Drp1/Middle.jpg]

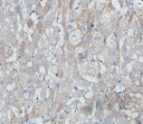

Supplement: Supplementary file 1 [file DataSheet1.ZIP › FigShares/FigShares/Figure 10/Fig 9H. Drp1/Model.jpg]

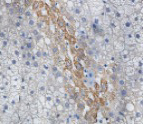

Supplement: Supplementary file 1 [file DataSheet1.ZIP › FigShares/FigShares/Figure 10/Fig 9H. Drp1/Positive.jpg]

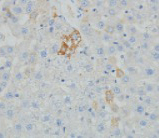

Supplement: Supplementary file 1 [file DataSheet1.ZIP › FigShares/FigShares/Figure 10/Fig 9H. Fis/Control.jpg]

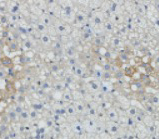

Supplement: Supplementary file 1 [file DataSheet1.ZIP › FigShares/FigShares/Figure 10/Fig 9H. Fis/High.jpg]

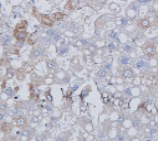

Supplement: Supplementary file 1 [file DataSheet1.ZIP › FigShares/FigShares/Figure 10/Fig 9H. Fis/Low.jpg]

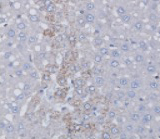

Supplement: Supplementary file 1 [file DataSheet1.ZIP › FigShares/FigShares/Figure 10/Fig 9H. Fis/Middle.jpg]

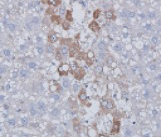

Supplement: Supplementary file 1 [file DataSheet1.ZIP › FigShares/FigShares/Figure 10/Fig 9H. Fis/Model.jpg]

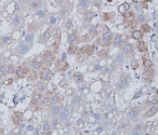

Supplement: Supplementary file 1 [file DataSheet1.ZIP › FigShares/FigShares/Figure 10/Fig 9H. Fis/Positive.jpg]

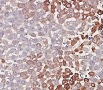

Supplement: Supplementary file 1 [file DataSheet1.ZIP › FigShares/FigShares/Figure 10/Fig 9H. Mfn1/Control.jpg]

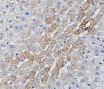

Supplement: Supplementary file 1 [file DataSheet1.ZIP › FigShares/FigShares/Figure 10/Fig 9H. Mfn1/High.jpg]

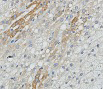

Supplement: Supplementary file 1 [file DataSheet1.ZIP › FigShares/FigShares/Figure 10/Fig 9H. Mfn1/Low.jpg]

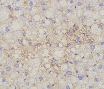

Supplement: Supplementary file 1 [file DataSheet1.ZIP › FigShares/FigShares/Figure 10/Fig 9H. Mfn1/Middle.jpg]

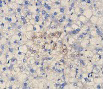

Supplement: Supplementary file 1 [file DataSheet1.ZIP › FigShares/FigShares/Figure 10/Fig 9H. Mfn1/Model.jpg]

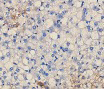

Supplement: Supplementary file 1 [file DataSheet1.ZIP › FigShares/FigShares/Figure 10/Fig 9H. Mfn1/Positive.jpg]

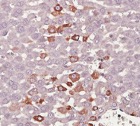

Supplement: Supplementary file 1 [file DataSheet1.ZIP › FigShares/FigShares/Figure 10/Fig 9H. Mfn2/Control.jpg]

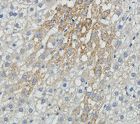

Supplement: Supplementary file 1 [file DataSheet1.ZIP › FigShares/FigShares/Figure 10/Fig 9H. Mfn2/High.jpg]

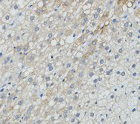

Supplement: Supplementary file 1 [file DataSheet1.ZIP › FigShares/FigShares/Figure 10/Fig 9H. Mfn2/Low.jpg]

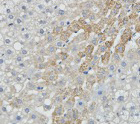

Supplement: Supplementary file 1 [file DataSheet1.ZIP › FigShares/FigShares/Figure 10/Fig 9H. Mfn2/Middle.jpg]

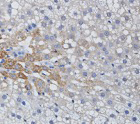

Supplement: Supplementary file 1 [file DataSheet1.ZIP › FigShares/FigShares/Figure 10/Fig 9H. Mfn2/Model.jpg]

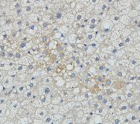

Supplement: Supplementary file 1 [file DataSheet1.ZIP › FigShares/FigShares/Figure 10/Fig 9H. Mfn2/Positive.jpg]

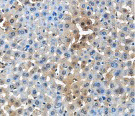

Supplement: Supplementary file 1 [file DataSheet1.ZIP › FigShares/FigShares/Figure 10/Fig 9H. Opa1/Control.jpg]

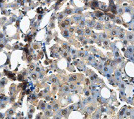

Supplement: Supplementary file 1 [file DataSheet1.ZIP › FigShares/FigShares/Figure 10/Fig 9H. Opa1/High.jpg]

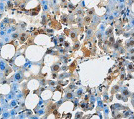

Supplement: Supplementary file 1 [file DataSheet1.ZIP › FigShares/FigShares/Figure 10/Fig 9H. Opa1/Low.jpg]

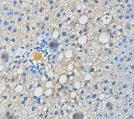

Supplement: Supplementary file 1 [file DataSheet1.ZIP › FigShares/FigShares/Figure 10/Fig 9H. Opa1/Middle.jpg]

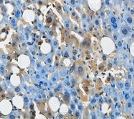

Supplement: Supplementary file 1 [file DataSheet1.ZIP › FigShares/FigShares/Figure 10/Fig 9H. Opa1/Model.jpg]

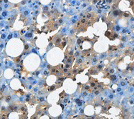

Supplement: Supplementary file 1 [file DataSheet1.ZIP › FigShares/FigShares/Figure 10/Fig 9H. Opa1/Positive.jpg]

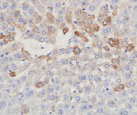

Supplement: Supplementary file 1 [file DataSheet1.ZIP › FigShares/FigShares/Figure 10/Fig 9H. Parkin/Control.jpg]

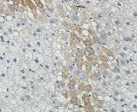

Supplement: Supplementary file 1 [file DataSheet1.ZIP › FigShares/FigShares/Figure 10/Fig 9H. Parkin/High.jpg]

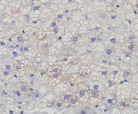

Supplement: Supplementary file 1 [file DataSheet1.ZIP › FigShares/FigShares/Figure 10/Fig 9H. Parkin/Low.jpg]

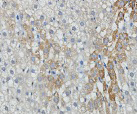

Supplement: Supplementary file 1 [file DataSheet1.ZIP › FigShares/FigShares/Figure 10/Fig 9H. Parkin/Middle.jpg]

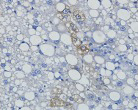

Supplement: Supplementary file 1 [file DataSheet1.ZIP › FigShares/FigShares/Figure 10/Fig 9H. Parkin/Model.jpg]

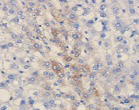

Supplement: Supplementary file 1 [file DataSheet1.ZIP › FigShares/FigShares/Figure 10/Fig 9H. Parkin/Positive.jpg]

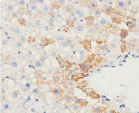

Supplement: Supplementary file 1 [file DataSheet1.ZIP › FigShares/FigShares/Figure 10/Fig 9H. PINK1/Control.jpg]

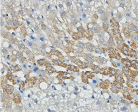

Supplement: Supplementary file 1 [file DataSheet1.ZIP › FigShares/FigShares/Figure 10/Fig 9H. PINK1/High.jpg]

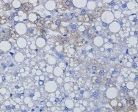

Supplement: Supplementary file 1 [file DataSheet1.ZIP › FigShares/FigShares/Figure 10/Fig 9H. PINK1/Low.jpg]

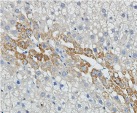

Supplement: Supplementary file 1 [file DataSheet1.ZIP › FigShares/FigShares/Figure 10/Fig 9H. PINK1/Middle.jpg]

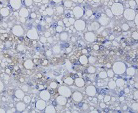

Supplement: Supplementary file 1 [file DataSheet1.ZIP › FigShares/FigShares/Figure 10/Fig 9H. PINK1/Model.jpg]

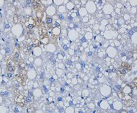

Supplement: Supplementary file 1 [file DataSheet1.ZIP › FigShares/FigShares/Figure 10/Fig 9H. PINK1/Positive.jpg]

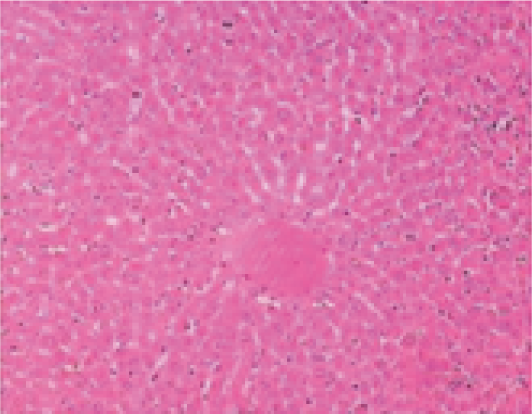

Supplement: Supplementary file 1 [file DataSheet1.ZIP › FigShares/FigShares/Figure 5/Fig 4A. control.tif]

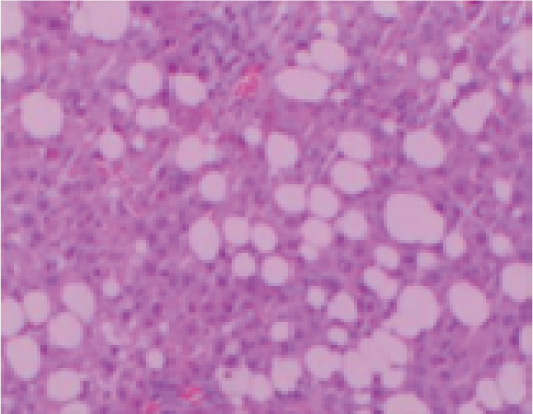

Supplement: Supplementary file 1 [file DataSheet1.ZIP › FigShares/FigShares/Figure 5/Fig 4A. model.tif]

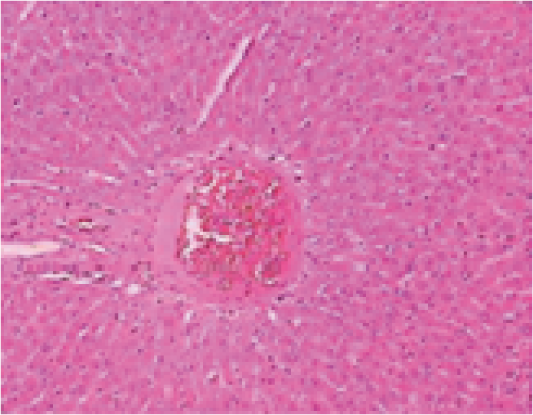

Supplement: Supplementary file 1 [file DataSheet1.ZIP › FigShares/FigShares/Figure 5/Fig 4B. control.tif]

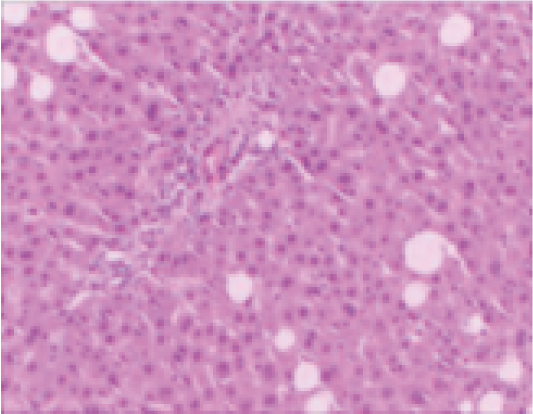

Supplement: Supplementary file 1 [file DataSheet1.ZIP › FigShares/FigShares/Figure 5/Fig 4B. high-dose.tif]

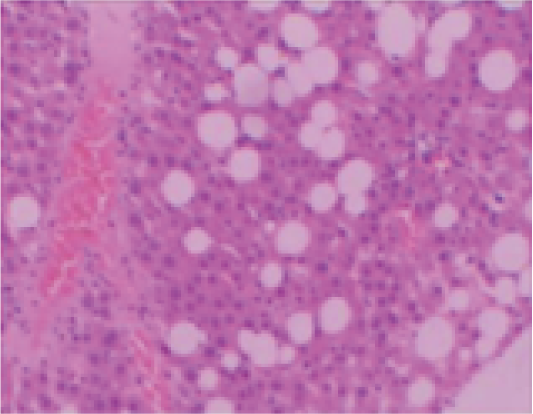

Supplement: Supplementary file 1 [file DataSheet1.ZIP › FigShares/FigShares/Figure 5/Fig 4B. low-dose.tif]

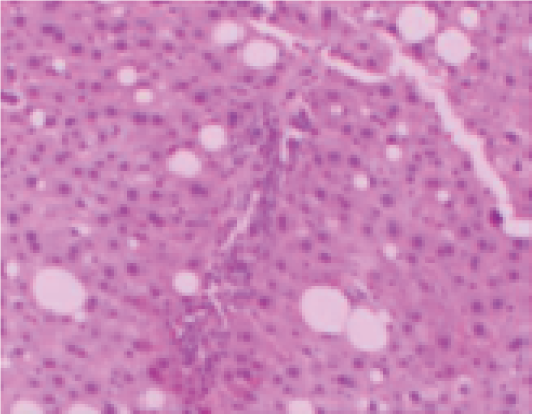

Supplement: Supplementary file 1 [file DataSheet1.ZIP › FigShares/FigShares/Figure 5/Fig 4B. middle-dose.tif]

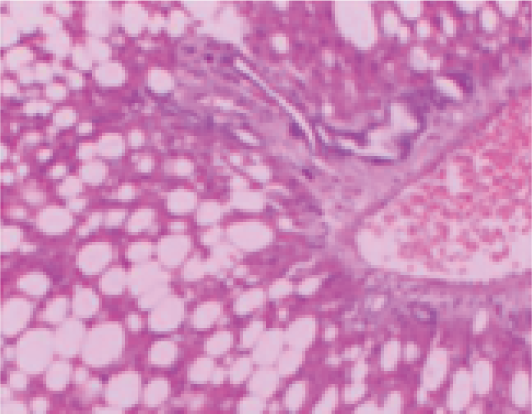

Supplement: Supplementary file 1 [file DataSheet1.ZIP › FigShares/FigShares/Figure 5/Fig 4B. model.tif]

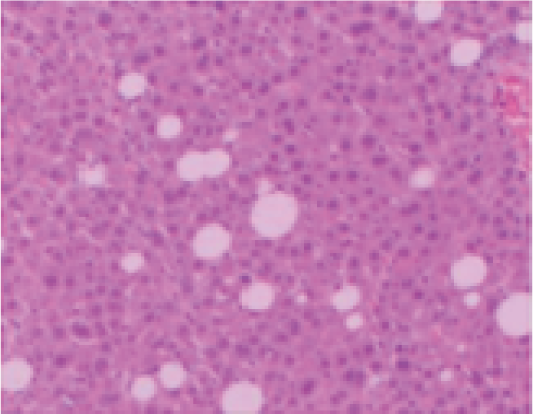

Supplement: Supplementary file 1 [file DataSheet1.ZIP › FigShares/FigShares/Figure 5/Fig 4B. positive.tif]

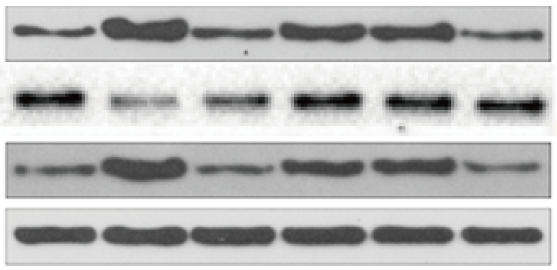

Supplement: Supplementary file 1 [file DataSheet1.ZIP › FigShares/FigShares/Figure 7/Fig 6D.tif]

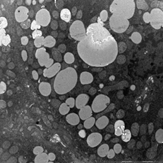

Supplement: Supplementary file 1 [file DataSheet1.ZIP › FigShares/FigShares/Figure 9/Fig 8A. model.tif]

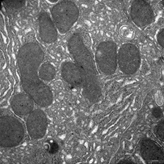

Supplement: Supplementary file 1 [file DataSheet1.ZIP › FigShares/FigShares/Figure 9/Fig 8A. control.tif]

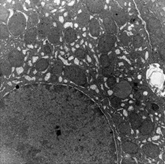

Supplement: Supplementary file 1 [file DataSheet1.ZIP › FigShares/FigShares/Figure 9/Fig 8A. high-dose.tif]

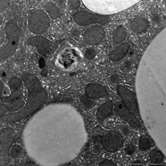

Supplement: Supplementary file 1 [file DataSheet1.ZIP › FigShares/FigShares/Figure 9/Fig 8A. low-dose.tif]

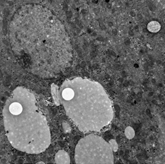

Supplement: Supplementary file 1 [file DataSheet1.ZIP › FigShares/FigShares/Figure 9/Fig 8A. middle-dose.tif]

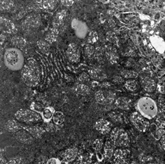

Supplement: Supplementary file 1 [file DataSheet1.ZIP › FigShares/FigShares/Figure 9/Fig 8A. positive.tif]

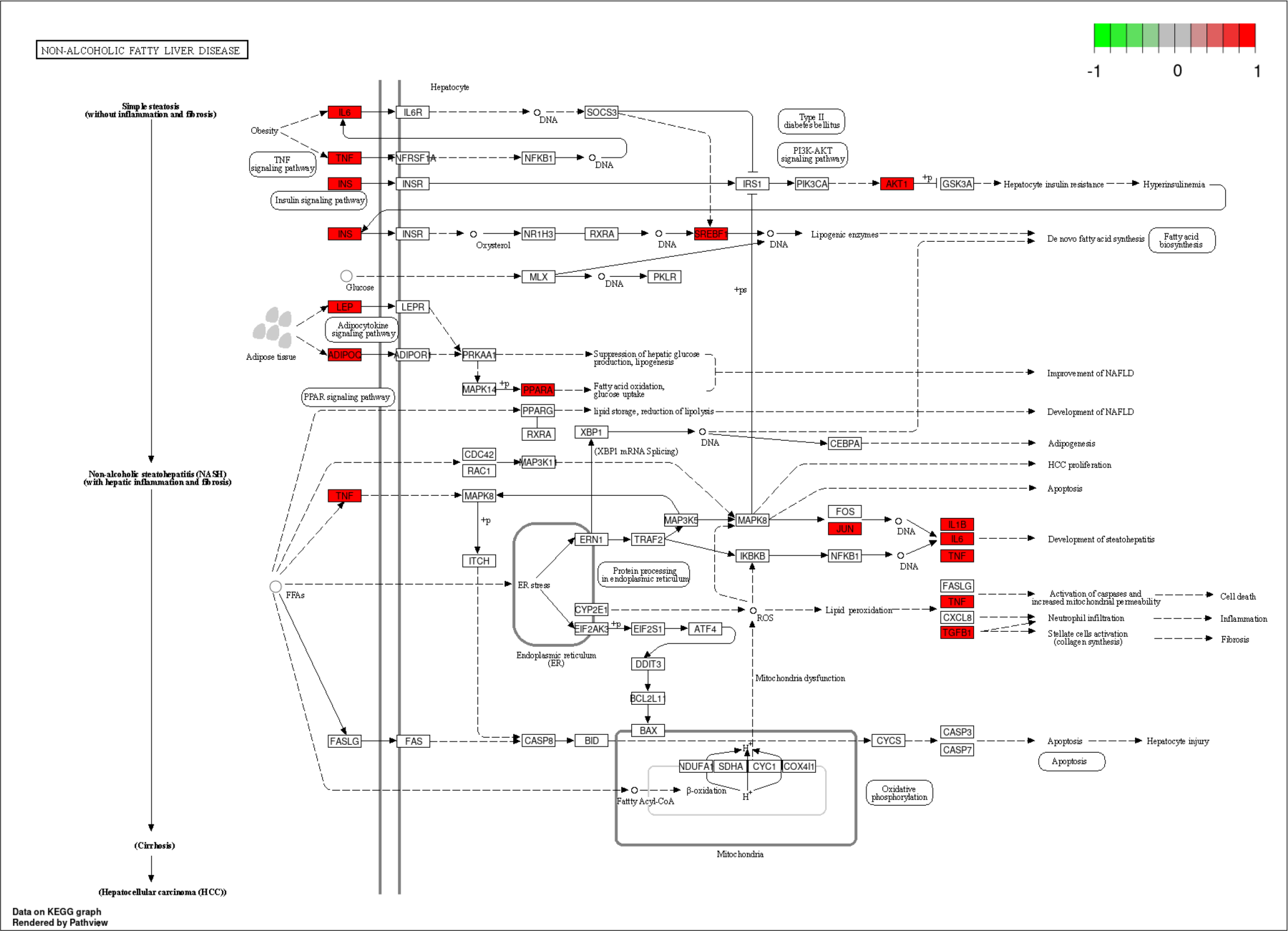

Supplement: Supplementary file 1 [file DataSheet1.ZIP › FigShares/FigShares/NAFLD KEGG pathway.tif]
